# Disrupting the CXCL12/CXCR4 axis disturbs the characteristics of glioblastoma stem-like cells of rat RG2 glioblastoma

**DOI:** 10.1186/1475-2867-13-85

**Published:** 2013-08-21

**Authors:** Chin-Cheng Lee, Jin-Huei Lai, Dueng-Yang Hueng, Hsin-I Ma, Yuan- Chiang Chung, Ya-yun Sun, Yih-Ju Tsai, Wen-Ben Wu, Chih-Li Chen

**Affiliations:** 1 Department of Pathology, Shin Kong Memorial Hospital, Taipei, Taiwan; 2 Department of Pathology, Taipei Medical University, Taipei, Taiwan; 3 Department of Optometry, Yuanpei University, Hsinchu, Taiwan; 4 Department of Neurological Surgery, Tri-Service General Hospital, National Defense Medical Center, Taipei, Taiwan; 5 Department of Surgery, Cheng Ching General Hospital, Chung- Kang Branch, Taichung City, Taiwan; 6 School of Medicine, Fu-Jen Catholic University, Taipei, Taiwan

**Keywords:** Glioblastoma stem-like cell (GSC), CXCR4, CXCL12, Angiogenesis, Invasiveness

## Abstract

**Background:**

Glioblastoma stem-like cells (GSC) have been shown to promote tumor growth, tumor-associated neovascularization, therapeutic resistance, and metastasis. CXCR4 receptors have been found involved in the proliferation, metastasis, angiogenesis, and drug-resistant characteristics of glioblastoma. However, the role of CXCR4 in modulating the stem-like cell properties of rat glioblastoma remains ambiguous.

**Methods:**

To explore the role of the CXCL12/CXCR4 axis in maintaining rat GSC properties, we disrupted the CXCR4 signaling by using small hairpin interfering RNA (shRNA). To investigate the role of the CXCL12/CXCR4 axis in maintaining rat GSC properties, we used a spheroid formation assay to assess the stem cell self-renewal properties. A western blot analysis and PCR arrays were used to examine the genes involved in proliferation, self-renewal, and cancer drug resistance. Finally, DNA content and flow cytometry, an immunohistochemical analysis, and methylcellulose colony formation, in vitro invasive and intracranial injection xenograft assays were employed to examine the disruptive effect of CXCR4 on the characteristics of GSCs of the RG2 cell line.

**Results:**

Disrupting CXCR4 inhibited the proliferation of RG2 cells both in vitro and in vivo. The spheroid formation assay indicated that CXCR4 was vital for the self-renewal of RG2 GSCs. Disrupting the CXCL12/CXCR4 pathway also reduced the expression of GSC cell markers, including Nestin, ABCG2, and musashi (Msi), and the expression of genes involved in regulating stem cell properties, including Oct4, Nanog, maternal embryonic leucine zipper kinase (MELK), MGMT, VEGF, MMP2, and MMP9.

**Conclusion:**

The chemokine receptor CXCR4 is crucial for maintaining the self-renewal, proliferation, therapeutic resistance, and angiogenesis of GSCs of rat RG2 glioblastoma.

## Background

Despite continual advances in surgical techniques, chemotherapy, and radiation regimens, the survival rate of patients with glioblastoma multiforme (GBM) remains bleak [1,2]. Recent studies have suggested that the progression of these brain tumors is driven by a small subpopulation of tumor cells known as cancer stem cells (CSCs), which can self-renew, proliferate, and generate a progeny of multiple neuroepithelial lineages [3]. Glioblastoma stem-like cells (GSCs) are critical promoters of tumor growth, tumor-associated neovascularization, therapeutic resistance, and metastasis, [4-6] and are vital to an aggressively invasive phenotype of GBM [7]. These studies have suggested that targeting GSCs can reduce tumor recurrence and substantially enhance GBM treatment.

CXCR4, a member of the large family of 7-transmembrane domain receptors, is coupled with heterotrimeric Gi proteins and activated by its ligand CXCL12 (SDF-1). Recent studies have indicated that the CXCL12/CXCR4 axis regulates tissue-specific stem cell proliferation, survival, and homing [8,9]. In addition, CXCR4 is the most common chemokine receptor expressed in cancer cells, including breast, pancreatic, and prostate cancers, and GBM [10,11]. Recently, CXCR4 overexpression has been detected in several CSCs, including GSC [8,12]. Studies have also demonstrated that the activation of CXCR4 by CXCL12 stimulates a specific and significant proliferative response in GSCs, but not in differentiated tumor cells [13]. However, the exact role of and mechanisms by which the CXCL 12/CXCR4 axis in GSCs promotes tumor proliferation and tumor-associated neovascularization remain ambiguous, and corresponding therapeutic treatments have yet to be identified.

Rat RG2 glioblastoma, which has a highly invasive growth pattern, is an effective GBM model that has been used in various preclinical studies to evaluate changes in vascular permeability [14]. After using the RG2 model, our findings indicated that the CXCL12/CXCR4 axis conveyed signals by using the AKT, and Erk pathways, demonstrating that CXCR4 contributes to the proliferation, but not the invasiveness, of in vitro RG2. Disrupting the CXCR4 impaired the drug resistance if RG2 and the self-renewal properties of in vitro GSCs, but it did not affect in vivo tumorigenesis. Furthermore, we observed changes in the levels of several molecules involved in the self-renewal, proliferation, drug resistance, and vascularization of GSCs that resulted from a decrease in the level of CXCR4. Our data suggest that CXCR4 modulates the progress of glioblastoma by maintaining the properties of GSCs.

## Results and discussion

### Disrupting CXCR4 abrogated the SDF-1/CXCR4 axis signal transduction pathways

We investigated the correlation between CXCR4 levels and clinical pathological statuses; the results indicated that a high level of CXCR4 was associated with malignant tumors (Additional file 1: Figure S1, Additional file 2: Table S1), which was consistent with the reports of previous studies. These findings suggest that CXCR4 plays a role in the progress of primary tumors. To investigate the role of CXCR4 in tumor progression, we screened the level of CXCR4 of several glioblastoma cell lines derived from humans, mice, and rats. Of these glioma cell lines, RG2, a rat glioblastoma cell line, was selected because it exhibits a high level of CXCR4 expression, highly invasive growth, and similar invasion patterns to human gliomas [15,16].

**Figure 1:**
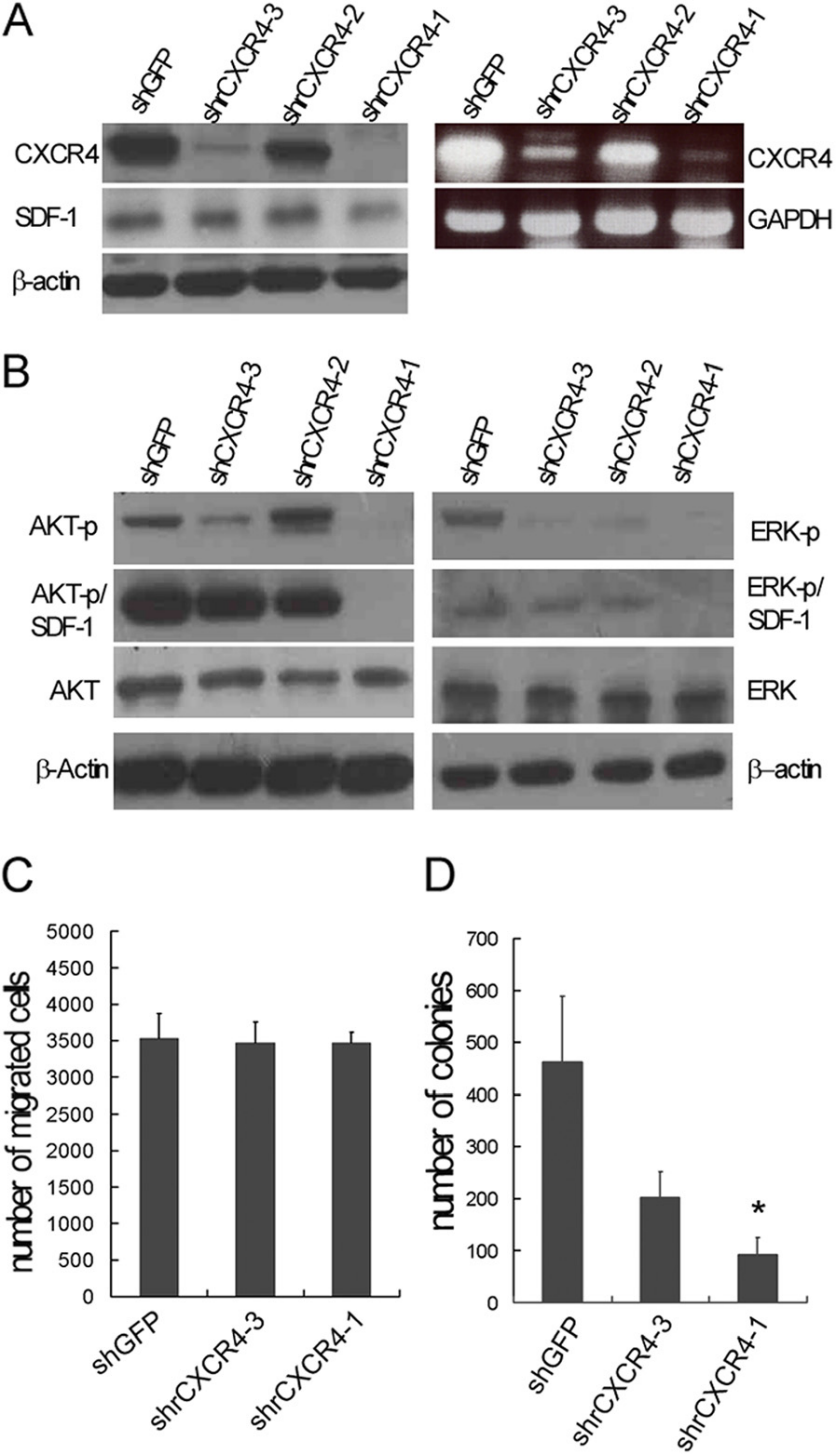
**Disruption of CXCR4 abrogated signal transduction pathways and the proliferation of rat glioblastoma, RG2. (A)** Representatives of combined individual clones with similar residual levels of CXCR4 from the rat glioblastoma cell line (RG2) with disrupted expression of CXCR4 by shRNA. Both western blotting and RT-PCR revealed decreased levels of CXCR4 in shrCXCR4-1 and shrCXCR4-3 clones, compared to the mock shGFP. **(B)** Disruption of CXCR4 impaired the phosphorylation of AKT and ERK without (AKT-p, ERK-p) or with induction of SDF1α (AKT-p/SDF-1, ERK-p/SDF-1α). **(C)** In vitro transwell assay indicated that inhibiting CXCR4 did not affect the invasiveness of RG2. **(D)** Methylcellulose colony formation assays indicated that the disruption of CXCR4 reduced the number of colonies. As shown was representative of similar results from two independent experiments.

Short-hairpin (shRNA)-containing plasmids that target the nucleotides of CXCR4 and control sequences of GFP were separately introduced into RG2 cell lines. The residual CXCR4 expression of selected clones was determined using western blotting and PCR. Two to three separate clones that possessed similar residual levels of CXCR4 were pooled together. Clones that possessed varying residual CXCR4 expressions and controls were designated shrCXCR4-3 (< 50%), shrCXCR4-1 (< 20%), and shGFP, and were chosen for further characterization (Figure 1A). The SDF-1 levels of the selected clones remained unchanged (Figure 1A, SDF-1). To investigate how disrupting CXCR4 expression affects the signal transduction pathway [17,18], cells were harvested after being treatment using or not using SDF-1, and the levels of phosphorylated ERK and AKT were determined. The results indicated that interrupting CXCR4 reduces both AKT and ERK phosphorylation in a dose-dependent fashion (Figure 1B). The methylcellulose colony formation and Boyden chamber invasive assays revealed that the SDF-1/CXCR4 axis is required for cell growth but not for the in vitro invasiveness of RG2 (Figure 1C, D). Subcutaneous injections of shGFP and shrCXCR4-1 into NOD-SCID mice revealed that disrupting CXCR4 impaired the proliferation of glioblastoma but not in vivo tumorigenesis (Additional file 3: Figure S2).

### Disrupting CXCR4 expression impaired sphere formation in glioblastoma stem-like cells (GSCs) of RG2

**Figure 2:**
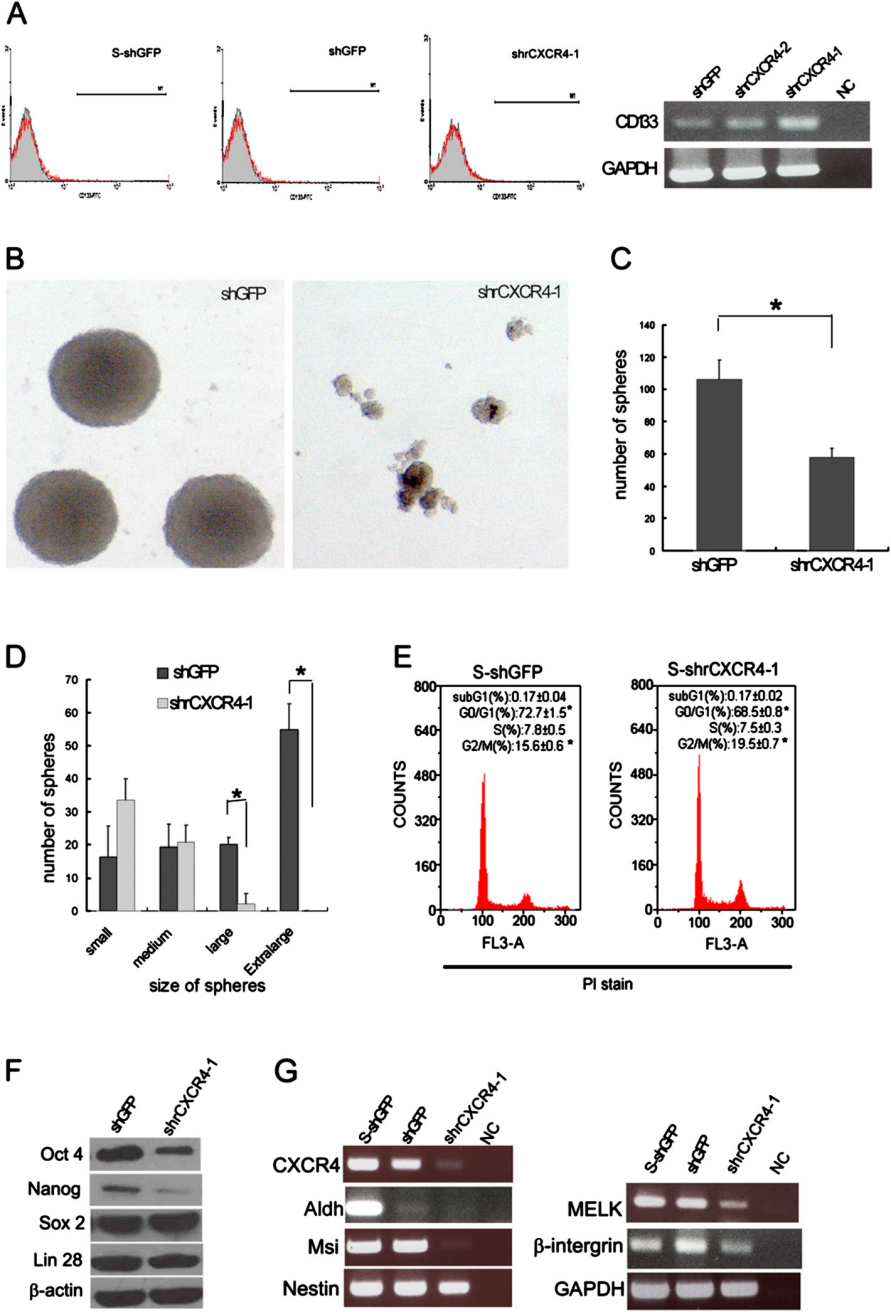
**CXCR4 is required by glioblastoma cell lines for maintaining self-renewal characteristics of cancer stem-like cells. (A)** Flow cytometry using anti-CD133 and RT-PCR indicated that RG2 had a low level of CD133. **(B)** Spheroid formation of the control shGFP and shrCXCR4 RG2 after culture in an ultralow dish for 10 d. Quantitative analysis showed a reduced number **(C)** and size **(D)** of spheres derived from the shrCXCR4 RG2 clone. Similar results were obtained from 2 independent experiments, and each experiment was performed in triplicate. **(E)** Histograms of cell cycle analysis showed that the deficiency of CXCR4 caused the reduction in G1 and the increase of G2/M populations. After being cultured in an ultralow dish 7d, the spheres derived from shGFP and shrCXCR4-1 were collected and trypsinized, and cell suspensions were subjected to PI staining. Comparing the cell cycle profiles revealed that the percentage of G2/M populations within cells collected from shrCXCR4-1 spheres (S-shrCXCR4, 19.5 ± 0.7%) was higher than those derived from shGFP (S-shGFP, 15.5 ± 0.6%) (*P < 0.05). By contrast, the percentage of G1 population within cells collected from shrCXCR4-1 (68 ± 0.8%) was lower than those from shGFP (72 ± 1.5%, *P < 0.05). **(F)** Western blot analysis showed that disrupting CXCR4 reduced the level of stem-cell-associated genes, including Oct4 and Nanog. **(G)** RT-PCR assay indicated that the expression of several genes, including stem-cell markers, Aldh, Nestin, Msi, and the proliferation promoting gene MELK increased in the control clone after forming spheroids, but decreased in the shrCXCR4-1 RG2. The shown were one of the similar results from 2 independent experiments.

Several studies have indicated that CD133+ or CD133- glioma cells have a stem-like cell population and can cause tumors [19,20]. We investigated the CD133 level of rat RG2 glioblastoma; the flow cytometry and RT-PCR results showed that the expression of CD133 in RG2 was low (Figure 2A). To investigate the role of CXCR4 in regulating the characteristics of CSCs, we tested sphere formation by using an ultralow plate system and a modified plate-well designed to test self-renewal properties by preventing cell attachment and differentiation. The result showed that the shrCXCR4-1 RG2 significantly lost its ability to form spheres (Figures 2B-D). We performed cell cycle analysis to determine whether the reduction number and sphere size were caused by the increase of apoptotic cells or the reduction in proliferation. As shown in Figure 2E, the percentage of G2/M populations within cells collected from shrCXCR4-1 spheres was higher (S-shrCXCR4, 19.5 ± 0.7%) than those from shGFP spheres (S-shGFP, 15.5 ± 0.6%), but the apoptotic population remained comparable (S-shGFP: 0.17 ± 0.02% vs. S-shrCXCR4-1: 0.17 ± 0.05%). By contrast, the percentage of G1 populations within the cells collected from shrCXCR4-1 (68 ± 0.8%) was lower than those from shGFP (72 ± 1.5%) (Figure 2E). This observation indicates that the reduction number and sphere size may be due to the reduction in proliferation. However, the in vivo data showed that disruption of CXCR4 impaired proliferation and increased apoptosis of RG2 glioblastoma (Additional file 3: Figure S2). We tested the levels of various transcription factors, including Oct4 [21], Nanog [22], and Sox2 [23], which are involved in the self-renewal of GSCs and the expression of maternal embryonic leucine zipper kinase (MELK) [24], and associated with GSC proliferation and the expression of GSC markers such as musashi (Msi), Nestin [25], and Aldh [3]. The results indicated that disrupting the CXCR4 reduced the levels of Oct4, Nanog, and the expression of Msi and MELK, and slightly reduced the expression of β-intergrin, Nestin and Aldh; the level of Sox2 and Lin 28 remained unchanged (Figures 2E, F). This indicates that the CXCL12/CXCR4 axis plays a significant role in maintaining the self-renewal properties of GSCs.

### Disrupting SDF-1/CXCR4 differentially increases the apoptosis of RG2 induced by cytotoxic chemotherapy

**Figure 3:**
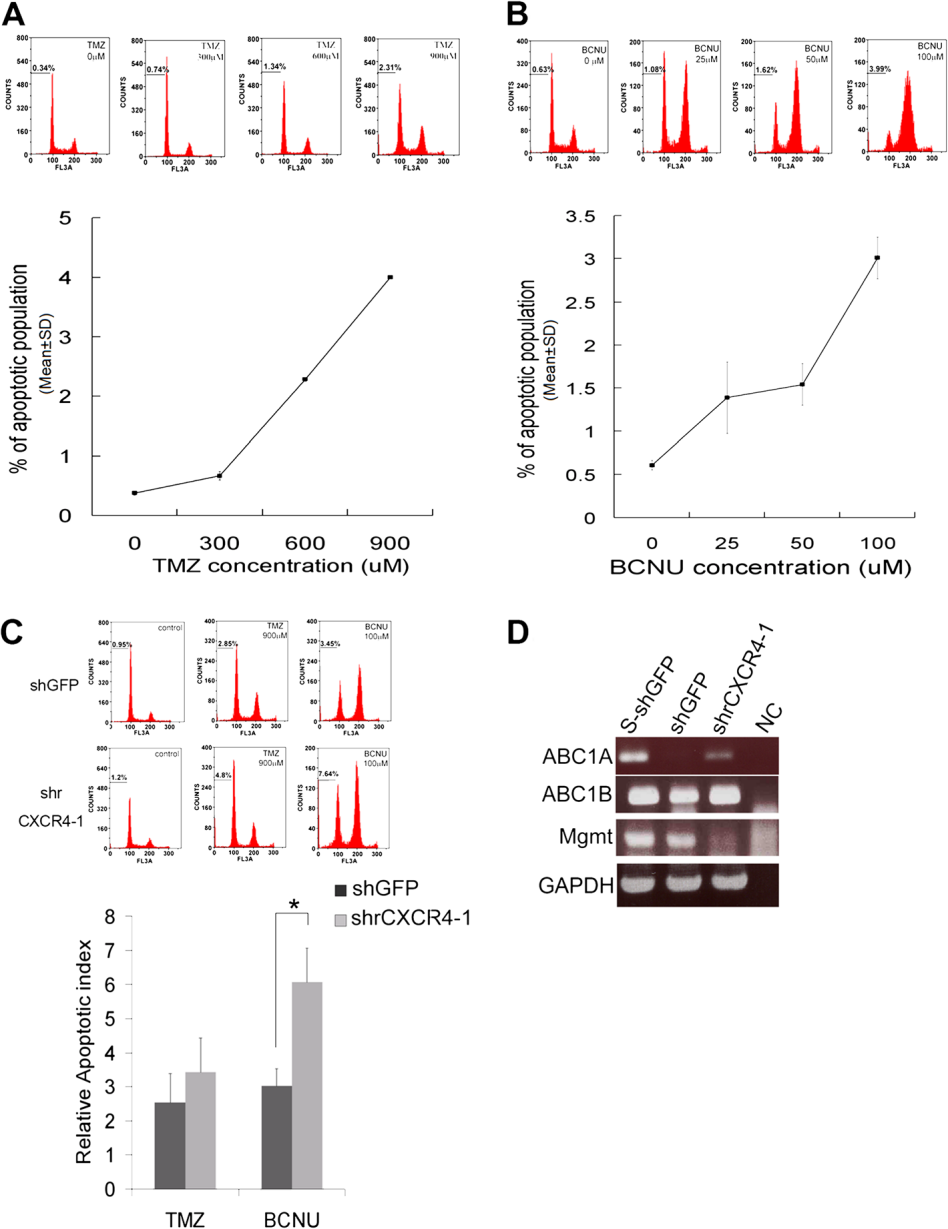
**Disrupting the CXCL12/CXCR4 axis increased the sensitivity of glioblastomas to antineoplastic drugs.** To investigate the effects of disruption of the CXCL12/CXCR4 axis on drug resistance, cells were plated at a density of 10 000 per well in a 12-well plate 1 d prior to drug treatment. TMZ or BCNU was added to achieve the indicated concentrations on the day of the experiment. After 24 h, cells were collected, fixed, stained with propidium iodide (PI) according to standard protocols, and analyzed. The histograms of flow cytometry showed that the RG2 cells were highly resistant to TMZ **(A)**, but only slightly resistant to BCNU **(B)**. Two independent experiments were done in triplicate and yielding similar results. As shown is the representative from one experiment. **(C)** Under normal-medium conditions, both shGFP and shrCXCR4 were treated with 900μM of TMZ or 100 μM of BCNU. PI staining revealed that the disruption of CXCR4 significantly increased the susceptibility of RG2 to BCNU, but only slightly increased its susceptibility to TMZ. The apoptotic index was defined as the fold of the apoptotic population of treated cells compared with the apoptotic population of vehicle-treated cells. Two independent clones of siGFP and shrCXCR4 were used to perform the experiments, yielding similar results. Each clone was used to perform 2 independent experiments in triplicate. (*t test, *P* < 0.05). **(D)** RT-PCR indicated that disrupting CXCR4 disturbed the expression of genes associated with drug resistance in GSCs.

GSCs are characterized by drug resistance. To test how disturbing CXCR4 affects the drug resistance and cytotoxic chemotherapy of glioblastoma, we used temozolomide (TMZ) [26] and 1, 3-bis (2-chloroethyl)-1-nitrosourea (BCNU) [26,27], which are alkylating drugs frequently used DNA to treat brain tumors. We first examined the optimal dosage of TMZ and BCNU for killing RG2 cell lines. The apoptotic effect of TMZ was not obvious until the concentration reached 900 μM, whereas BCNU exhibited an apoptotic effect at a concentration of 100 μM (Figures 3A, B). In normal medium conditions, both the shGFP and shrCXCR4 were treated using 900 μM of TMZ or 100 μM of BCNU. The apoptotic index was defined as the fold of the apoptotic population of treated cells compared with the apoptotic population of vehicle-treated cells. Disrupting the SDF-1/CXCR4 pathway only slightly increased the cytotoxic effect of TMZ; however, reducing CXCR4 expression significantly increased the cytotoxic effect of BCNU (Figure 3C). Several molecules have been implicated in the drug resistance of CSCs. In particular, MGMT has been suggested in both TMZ and BCNU resistance. We performed semi-quantitative RT-PCR to explore the expression of molecules implicated in the drug resistance of CSCs, including ABCb1A, ABCb1B [3], and MGMT [28]. The results showed that the expression of these molecules increased in cells isolated from the sphere formed by shGFP RG2. In addition, the expression of MGMT was higher in shGFP compared with shrCXCR4 RG2, whereas the level of ABCb1A and ABCb1B increased slightly in shrCXCR4-1, compared with the levels of these molecules in shGFP (Figure 3D).

### Disrupting the expression of CXCR4 impairs vascularization of xenografts derived from rat RG2 glioblastoma cells

**Figure 4:**
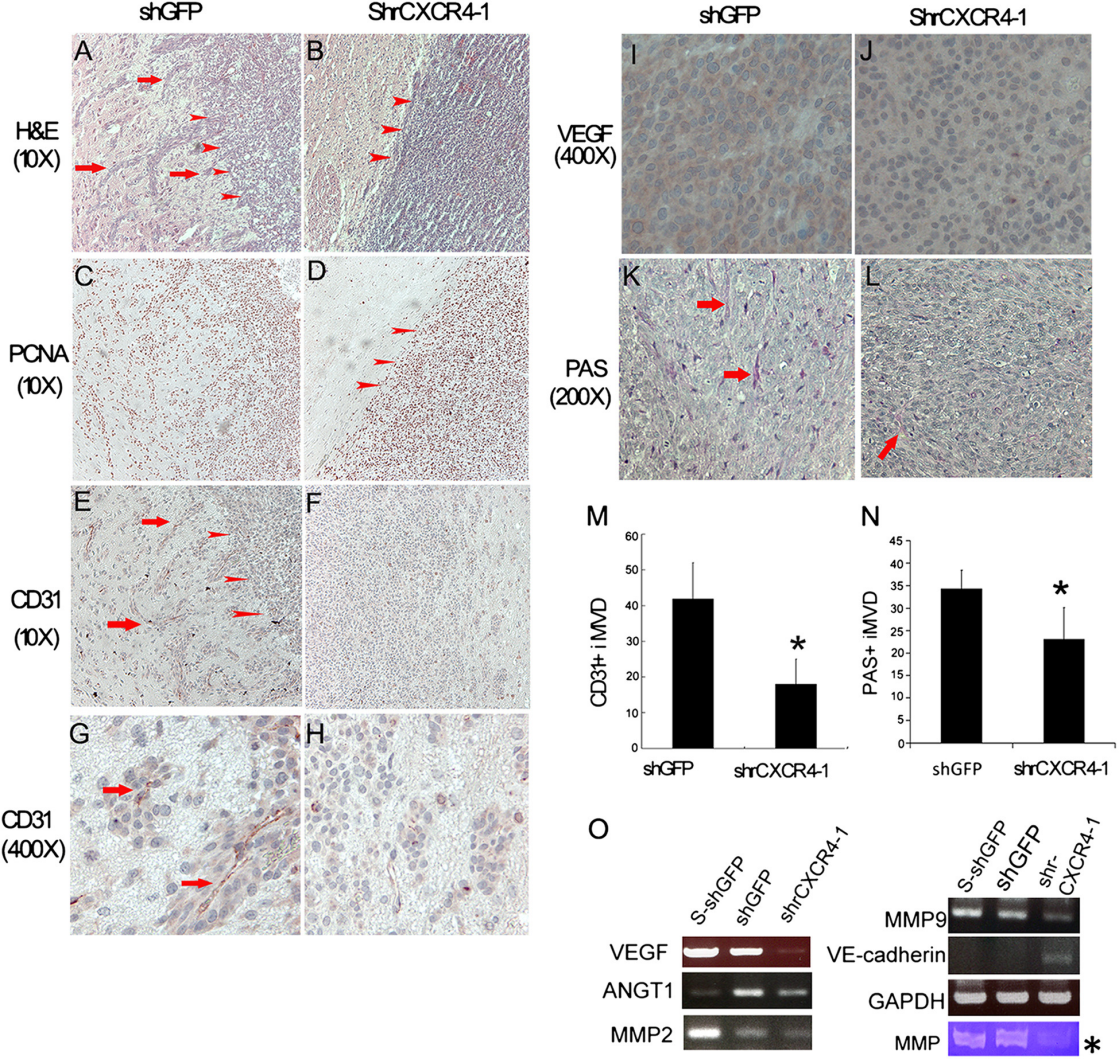
**Disruption of the CXCL12/CXCR4 axis impaired the neovascularization of rat RG2 glioblastoma in vivo. (A, B)** H&E staining of sections collected from orthografts showed more cells sprouting from the tumor core from the control shGFP RG2 **(A)** than from shrCXCR4 RG2 **(B)** (red arrow: sprouting RG2 cells). **(C, D)** Immunohistochemistry using anti-PCNA staining showed more proliferating cells spreading in the sections collected from grafts of shGFP RG2(C) than those from shrCXCR4 **(D)**. Immunohistochemistry using anti-CD31, an endothelium cell marker, showed more CD31-positive microvessels in the grafts derived from shGFP RG2 **(E, G)** than in those derived from shrCXCR4 **(F, H)**. The CD31 positive tubes were sprouting from the tumor core in the sections from the grafts of shGFPRG2. (red arrows, CD31 positive vessels surrounded by cancer cells). **(I, J)** The levels of VEGF were reduced in the grafts derived from shrCXCR4-1 RG2 **(I)**, compared to those from shGFP RG2 **(J)**. **(K, L)** PAS, a marker of basement membrane staining, indicated that more PAS-positive microvessels existed in tumors derived from shGFP **(K)** than in those derived from shrCXCR4-1 **(L)** (red arrow, PAS positive tubes). **(M, N)** Quantitative analysis showed that the density of CD31 positive **(M)** and PAS-positive **(N)** microvessels was higher in the tumor derived from shGFP. **(O)** RT-PCR indicated that disruption of CXCR4 reduced the expression of genes regulating angiogenesis, including VEGF, ANGT1, MMP2, and MMP9, but not VE-cadherin. Arrowheads, the boundary of the tumor core, *result from zymography. *t test, *P* < *0.05.*

Evidence indicates that the SDF-1/CXCR4 axis significantly contributes to intratumoral angiogenesis [29]. To explore the role of CXCR4 in regulating the vascularization of glioblastoma, either shrCXCR4-1, or shGFP were intracranially injected into NOD-SCID mice. In accordance with the subcutaneous xenografts, hematoxylin and eosin (H & E) staining indicated that disrupting the CXCR4 did not impair in vivo tumorgenesis, but the tumors derived from CXCR4-deficient cells were smaller than those derived from shGFP. The proliferating cells as indicated by PCNA were reduced in the xenografts derived from the CXCR4-deficient cell lines. However, more cells spread from the center of the tumor derived from the control cell lines (Figures 4A-D). After anti-CD31 staining, we observed that more CD31 positive microvessels sprouted from the tumor derived from control cells than those from CXCR4-deficient cell lines (Figures 4E-H). A quantitative analysis revealed that knocking down the expression of CXCR4 significantly reduced the intratumoral microvessel density (iMVD) (Figure 4M). The results also showed that CXCR4 deficiency led to a reduction in PAS-positive intratumoral vessel density (Figures 4K, L, and N). However, in the shrCXCR4-1 xenografts, the density of the PAS-positive vessel was 20% (PAS: CD31: 25 vs. 18) higher compared with the CD31-positive vessel. This indicates that alternative mechanisms may cause vascularization after CXCR4 disruption. To explore the molecules involved in angiogenesis, we performed RT-PCR to detect the expression of VEGF, VE-cadherin [30,31], angiopoietin 1 (AGNT1) [32], MMP2 [33], and MMP9 [34], and of shrCXCR4 and shGFP RG2 cells. The result showed that disrupting CXCR4 impaired the expression of VEGF, AGNT1, MMP2, and MMP9, whereas it increased the level of VE-cadherin, which is a major endothelial adhesion molecule that controls cellular junctions and blood vessel formation (Figure 4O). In addition, gelatin zymography showed reduced activity in the matrix metalloproteinases (MMPs), which are molecules involved in vascularization (Figure 4O*). This suggests that the CXCL12/CXCR4 axis is critical to the vascularization of glioblastoma.

## Discussion

In accordance with previous studies, we demonstrated that the CXCL12/CXCR4 axis plays a substantial role in regulating the proliferation, drug resistance, and neovascularization of glioblastoma. However, the current study is the first to show that CXCR4 plays a critical role in maintaining the CSC characteristics of rat RG2 glioblastoma. The results show that disrupting CXCR4 selectively reduces the level of Oct4, Nanog, and MELK, but not that of Lin28 and Sox2. This suggests that the reduction of Oct4, Nanog, and MELK resulted from the destruction of CXCR4 signaling, rather than the change of cell fate. The data suggest that CXCL12/CXCR4 executes this function through the ERK and AKT pathways, regulating the expression of Oct4, Nanog, and MELK [35].

The current study was the first to show that RG2 has a high capacity for forming spheroids, exhibiting high levels of the molecules involved in maintaining the properties of stem-like cells. In addition, the expression of the ALDH, ABCG2, CXCR4, Nestin, and Msi, CSC markers was elevated in the cells of spheroids derived from shGFP (Figure 4). This indicates that RG2 cells have a cancer stem-like population. Although the results show a level of CD133 in RG2, this challenges CD133 as an obligate CSC marker [19]. The findings demonstrated that RG2 is highly prolific, invasive, and drug resistant. Thus, we speculate that RG2 has a stem-cell like population, comprising a low CD133 levels that may represent certain types of human glioblastoma.

Scholars have suggested that GBM GSCs that express high levels of MGMT are the key components that cause resistance to therapy. TMZ is the primary chemotherapeutic molecule used to treat GBM. The DNA adducts generated by TMZ are removed by MGMT [36,37]. In the current study, we determined that RG2 cells, similar to GSCs, express MGMT and are highly resistant to TMZ. Disrupting CXCR4 resulted in the impaired resistance of RG2 to both TMZ and BCNU, suggesting that impaired resistance to these molecules is caused by the reduced expression of MGMT. However, the knockdown of CXCR4 has distinct effects on the expression of ABC-transporters ABCb1B and ABCb1A. The results indicate that CXCR4 plays distinct roles in modulating the expression of molecules implicated in the expulsion of toxic agents.

Scherer suggested that the perivascular space contained specialized properties that are critical to maintaining and spreading glioblastoma [38]. Studies have found that these specialized properties include maintaining a stem-cell-like phenotype in glioblastoma cells localized in the niche region [39,40]. Therefore, the perivascular space is thought to contribute to tumor growth and therapeutic resistance. Rao et al. and other scholars determined that targeting the CXCL12/CXCR4 pathway can abrogate a specialized trophic function of GBM-associated vasculature that contributes to brain tumor growth [41]. Furthermore, Farin et al. observed that GBM cells travel along blood vessels and pause at select vascular branch points to proliferate [42]. In agreement with their findings, our results from the intracranial xenograft model reveal that disrupting the CXCL12/CXCR4 pathway reduces both the vessels that sprout from the tumor core and the intratumoral microvessel density. A decreased number of satellites was also observed in the grafts from shrCXCR4-1 RG2 (Additional file 4: Figure S3), indicating the decreased invasiveness of shrCXCR4 RG2. The current study also showed that the CXCL12/CXCR4 axis may regulate angiogenesis by regulating the expression of VEGF, ANGT1, and MMP9, which are major contributors to angiogenesis. However, the results of PAS staining and the increased expression of VE-cadherin indicate that blocking the CXCR4 pathway may not completely abrogate vascularization [31]. This may explain why RG2 continued to generate tumors despite the disrupted expression of CXCR4. The in vitro invasiveness assay suggested that the CXCL12/CXCR4 pathway is not vital to the migration of RG2 glioblastoma. In vivo observations indicated that the role of the CXCL-12/CXCR4 axis in the local recurrence or invasiveness of glioblastoma may be vital in modulating the ability of glioblastoma cells to proliferate and induce angiogenesis, but not to migrate [38,43,44].

## Conclusions

By using rat RG2 glioblastoma, we showed that disrupting the CXCL12/CXCR4 axis impairs the characteristics of GSCs. CXCR4 is known to be involved in the proliferation and angiogenesis of glioblastoma and in determining its invasiveness and resistance to drugs. In contrast to previous findings, we characterized the CXCL12/CXCR4 axis by using the RG2 cell line. The results suggest that CXCL12/CXCR4 is involved in the progress of glioblastoma, regulating the expression of the molecules associated with stem-cell properties. The importance of characterizing RG2 lies in the continual demand for experimental neuro-oncology animal models that can be used to assess the efficacy of new approaches for treating brain tumors. Although previous evidence has suggested that CXCL12/CXCR4 is an effective therapeutic target for glioblastoma, our findings elucidate the molecular mechanisms of CXCR4 involved in the progress of glioblastoma, additionally clarifying the properties of rat glioblastoma for use as an animal model in the treatment of brain tumors.

## Materials and methods

### Cell culture and transfection

RG2 cells (provided by the laboratory of Dr. Ma) were cultured in 90% Dulbecco’s modified Eagle’s medium that contained 10% fetal bovine serum, which was supplemented using 4 mM of L-glutamine adjusted to contain 1.5 g/L of sodium bicarbonate and 4.5 g/L of glucose, penicillin (100 U/mL), and streptomycin (100 mg/mL) (Invitrogen Life Technologies, Carlsbad, CA, USA). The cells were transfected as described in [45]. Cells at approximately 80% confluence in 3.5 cm culture plates (Falcon) were transfected with 8 μg of total DNA by using lipofectamine (Invitrogen Life Technologies) according to manufacturer protocols. Following transfection, the cells were selected using 600 μg/mL of G418 and individual colonies were picked up. The expanded colony was maintained in a selective medium that contained 300 μg/mL of G418. The expression and level of CXCR4 were determined using RT-PCR and western blotting, respectively. The CXCR4 deficient clones were designated shrCXCR4 and the control clones were designated shGFP.

### Intratumoral microvessel density (iMVD)

Depending on the size of the H & E section, 5 to 8 areas within the tumor were randomly selected for evaluation at 100X magnification. These areas were subsequently used to analyze the iMVD measurements at 200X magnification. The MVD was measured according to the Weidner method [46]. Each positive endothelial cell cluster of immunoreactivity that contacted the selected field was counted as a single vessel, including the morphologically identifiable vessels with lumens.

### Animals and generation of xenografts

We performed animal experiments in accordance with the Experimental Animal Management Ordinance approved by the Institutional Animal Care and Use Committee of the National Defense Medical Center of Taiwan. Each experimental group used 5–6 four- to eight-week-old female NOD-SCID mice. Each mouse was injected intracranially or subcutaneously with 2.5 × 10^6^ CXCR4-deficient cells (shrCXCR4 RG2) or the aforementioned control, shGFP RG2 [47]. After 2 months, the animals were anesthetized, and 4 μM paraffin-embedded sections of the brain or xenograft tissues were prepared for immunohistochemistry. The sections were stained using H & E and examined using a microscope for the presence of metastatic tumor foci (satellites).

### Western blotting

For the cell lysate preparation, the cells were washed using prechilled in phosphate buffered saline and lysed in an HI-RIPA buffer comprising 20 mM of Tris–HCl, pH 7.5; 150 mM of NaCL; 2 mM of EDTA; 1 mM of sodium fluoride; 0.5% Triton X-100; 0.5% sodium deoxycholate; 0.5% SDS; 10% glycerol; 1 mM of sodium orthovanadate; 1 mM of PMSF; and 1 mg/mL of aprotinin and leupeptin (freshly prepared). Protein assay, SDS PAGE, and western blotting were performed to conform to standard procedures. Additional file 5: Table S2 lists the antibodies and conditions used in western blot and immunohistochemistry.

### Plasmid of shRNA and reverse transcriptase polymerase chain reaction (RT-PCR)

Regarding the RNAi of CXCR4, we expressed the shRNA molecules targeted at sites beginning at Nucleotide 22. Additional file 6: Table S3 lists the oligonucleotides used to target the shRNA to the CXCR4, and the control GFP (synthesized by Integrated DNA Technologies, Coralville, IA, USA). The oligonucleotides were annealed and ligated into a pSilencer 2.1-U6 neo (Ambion, Austin, TX, USA), according to manufacturer directions. The constructs were sequentially verified.

Regarding PCR, 80% confluence of shGFP, shrCXCR4, and sphere shGFP (S-shGFP) cells were collected and immediately lysed using a Trizol reagent (Invitrogen Tech), and the total RNA was isolated. For each population, the first-strand DNA (cDNA) was formed using 5 μg of total RNA according to the Superscript III First Strand Synthesis System (Invitrogen Tech.). Additional file 6: Table S3 lists the sequences of primers used to amplify the indicated genes with PCR (MDbio Inc, Taipei, Taiwan). PCR was performed using 30 cycles of denaturation at 94°C, annealing at 55°C–60°C for 1 min, and elongating at 72°C for 1 min.

### Drug treatment and cell apoptosis assay

The cells were plated at a density of 10^5^ per well in a 12-well plate 1 d prior to the drug treatment. TMZ (Sigma) or BCNU (Bristol-Myers Squibb, Princeton, NJ, USA) was added to achieve the indicated concentrations on the day of the experiment. After 24 h, the cells were collected, fixed, stained using propidium iodide (PI) according to standard protocols, and analyzed using a Partec Cyflow ML flowcytometer (Partech GmBH, Münster, Germany). The relative percentage of cells in each cell-cycle compartment was estimated using Cell Quest Pro (BD Biosciences). The apoptotic index was defined as: the % of apoptotic cells treated using drugs at the indicated time intervals/the % of apoptotic cells treated using a vehicle at the indicated time intervals.

### Ultralow spheroid assay

The cells were cultured on a 10 cm ultralow plate at a density of 1000 per mL by using 10% FBS medium [48,49]. After 7 d, the cultures were collected in a 15 mL centrifuge tube and left standing for 3 min to precipitate spheres. The supernatant was discarded and the spheres were gently suspended using a 2 mL medium without FBS, then separately plated into a 12-well plate. Images were captured at 8X magnification. All the spheres were counted and their sizes was determined as follows: extralarge: diameter > 2 mm; large: diameter 1.5 mm–2 mm; medium: diameter 1.0 mm–1.5 mm; small: diameter < 1 mm.

### Statistical analysis

All data for the colony formation, invasion, iMVD, and proliferation assays were expressed using the stand error mean. The means between the two groups were compared using a two-tailed Student t test, and a *P* < 0.05 was considered statistically significant.

## Abbreviations

GSC: Glioblastoma stem cell; TMZ: Temozolomide; BCNU 1: 3-bis (2-chloroethyl)-1-nitrosourea; IHC: Immunohistochemistry; MMPs: Metalloproteinases; ShRNA: Short hairpin interfering RNA; MELK: Maternal embryonic leucine zipper kinase; Msi: Musashi.

## Competing interests

The authors declare that they have no competing interests.

## Authors’ contributions

LCC and SYY executed the western blotting, RT-PCR, and shRNA silence experiments and drafted the manuscript. LJH and HDY both participated in the animal experimental procedures. SYY and WWB also executed the MTT, zymography, and cell cycle analysis and invasion experiments. LCC, MHI, and CYC performed immunohistochemical analyses and examinations of the tissue samples. TYJ executed the cAMP assay. CLC supervised the project, making substantial contributions to the concept and design of the study, analyzing and interpreting the data, and writing the manuscript. All authors read and approved the final manuscript.

## Supplementary Material

Additional file 1: Figure S1 A high level of CXCR4 is associated with malignant brain tumors, but not with normal brain tissues. To investigate the correlation between CXCR4 levels and clinical pathological statuses, a primary brain tumor high-density (208 cores) tissue array of astrocytoma, glioblastoma, glioblastoma multiforme (GBM), and normal tissues was used to perform immunohistochemistry. The array contained triplicate cores per case: 60 cases of cancer and 9 cases of normal tissue. After CXCR4 staining, the tissue with a weak or no signal of CXCR4 was grouped in CXCR4 low (A, B) and the tissue with a high intensity of CXCR4 was grouped in CXCR4 high (C). The results indicated that a high level of CXCR4 was associated with malignant tumors (D). The pathological status also correlated with the level of CXCR4 (Additional file 2: Table S1). These findings suggest that CXCR4 plays a role in the progress of primary tumors. Click here for file

Additional file 2: Table S1 A high level of CXCR4 is associated with the pathological status of glioblastoma. Click here for file

Additional file 3: Figure S2 Disruption of CXCR4 results in RG2 proliferation deficiency and the increase of apoptosis in vivo. To explore the effect of CXCR4 disruption in the tumorigenesis of GSC in vivo, shrCXCR4-1 cells and shGFP cells were subcutaneously injected into NOD-SCID mice. After 21 d, both shrCXCR4 and shGFP cells grew to tumor mass. Xenografts derived from shrCXCR4 showed a tumor mass size that was smaller than those derived from shGFP (A, B). Immunohistochemistry using anti-PCNA, a proliferating cell marker, was performed to explore the proliferating cells of sections obtained from shGFP and shrCXCR4 xenografts. The PCNA-positive population of xenografts derived from shGFP was 70% and significantly dropped to 50% of those derived from shrCXCR4 (C, D). PCNA index: PCNA positive cells (brown)/hematoxylin positive cells (blue). The results showed that there were fewer proliferating cells in the xenografts derived from shrCXCR4-1 than those in the xenograft derived from shGFP. The apoptotic cells were revealed by TdT labeling by using TACS2 TdT-blue label in situ apoptosis detection kit (Travigen, Inc. Cat. 4811-30-K). The result showed that the apoptotic population of xenografts derived from shGFP was 30% and significantly increased to 40% of those derived from shrCXCR4 (C TdT labeling, D TdT labeling index). TdT labeling index: TdT label positive cells (blue)/nuclear fast red positive cells (red). This observation suggested that CXCR4 plays an essential role in the proliferation of glioblastoma cells. *t test, *P < 0.05.* Click here for file

Additional file 4: Figure S3 Disruption of CXCR4 markedly reduced the number of satellites, which were defined as tumor foci with blood vessel and away from the tumor core (TC). H&E staining revealed a reduced number of satellites (arrow) in the intracranial grafts from shrCXCR4-1 RG2 (B) as compared with those from shGFP RG2 (A). (C) Average of number of satellites per xenografts, indicating less number of satellites in the xenografts derived from shrCXCR4 RG2 as compared with those derived from GFP RG2. Number of satellites was counted as the average of 6 adjacent sections of each grafts. Representatives are the average number of satellites from 5 xenografts derived from shrCXCR4 or shGFP RG2. Click here for file

Additional file 5: Table S2 Listed antibodies for immunoblot blot and immunohistochemistry. Click here for file

Additional file 6: Table S3 Listed primers for RT-PCR. Click here for file
