# Supplementary figures and images for: Disrupting the CXCL12/CXCR4 axis disturbs the characteristics of glioblastoma stem-like cells of rat RG2 glioblastoma
Source: Cancer Cell Int. 2013 Aug 21;13:85. doi: 10.1186/1475-2867-13-85 (PMC3765790; doi:10.1186/1475-2867-13-85)

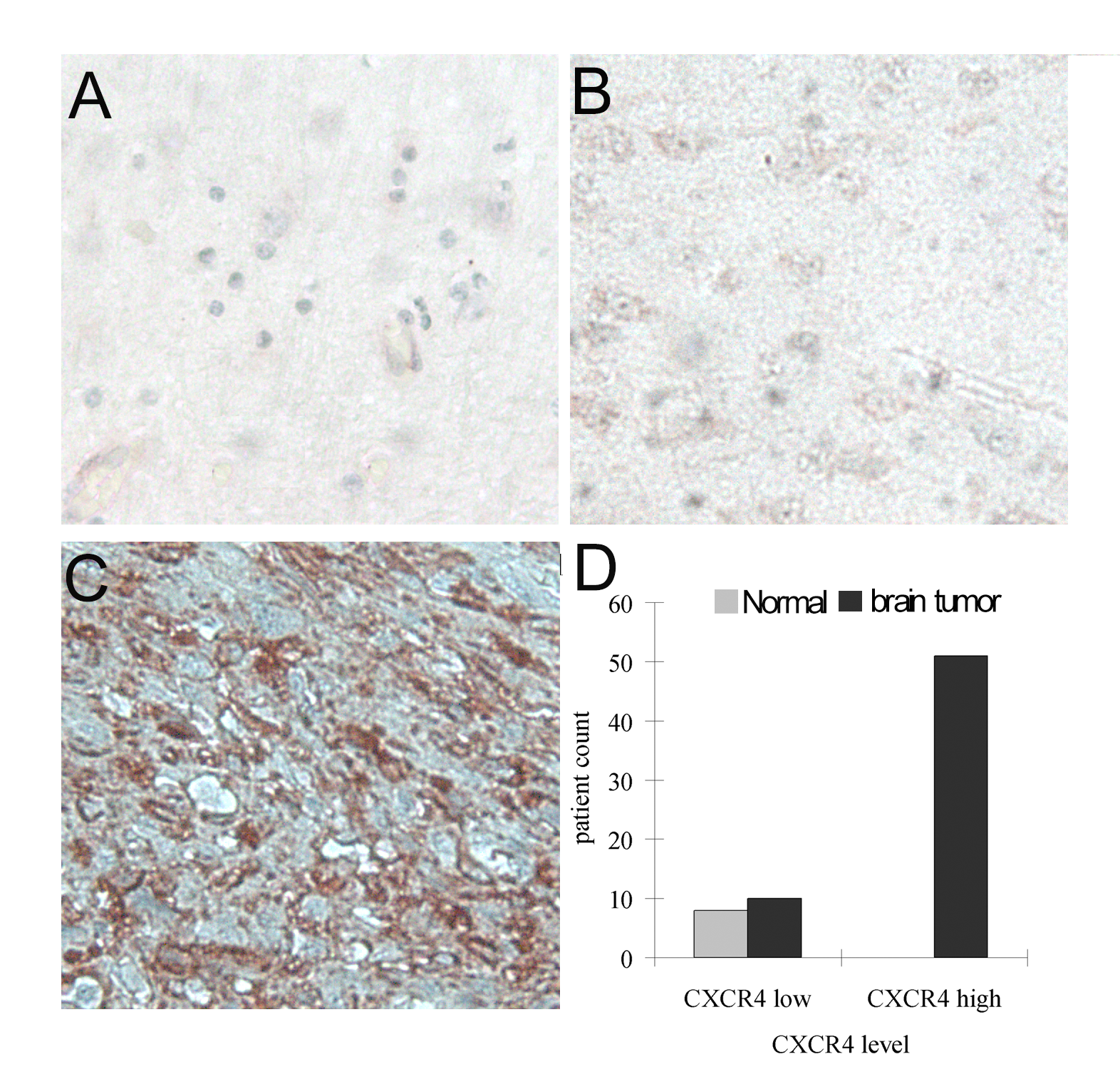

Supplement: Additional file 1: Figure S1 — A high level of CXCR4 is associated with malignant brain tumors, but not with normal brain tissues. To investigate the correlation between CXCR4 levels and clinical pathological statuses, a primary brain tumor high-density (208 cores) tissue array of astrocytoma, glioblastoma, glioblastoma multiforme (GBM), and normal tissues was used to perform immunohistochemistry. The array contained triplicate cores per case: 60 cases of cancer and 9 cases of normal tissue. After CXCR4 staining, the tissue with a weak or no signal of CXCR4 was grouped in CXCR4 low (A, B) and the tissue with a high intensity of CXCR4 was grouped in CXCR4 high (C). The results indicated that a high level of CXCR4 was associated with malignant tumors (D). The pathological status also correlated with the level of CXCR4 (Additional file 2: Table S1). These findings suggest that CXCR4 plays a role in the progress of primary tumors. [file 1475-2867-13-85-S1.tiff]

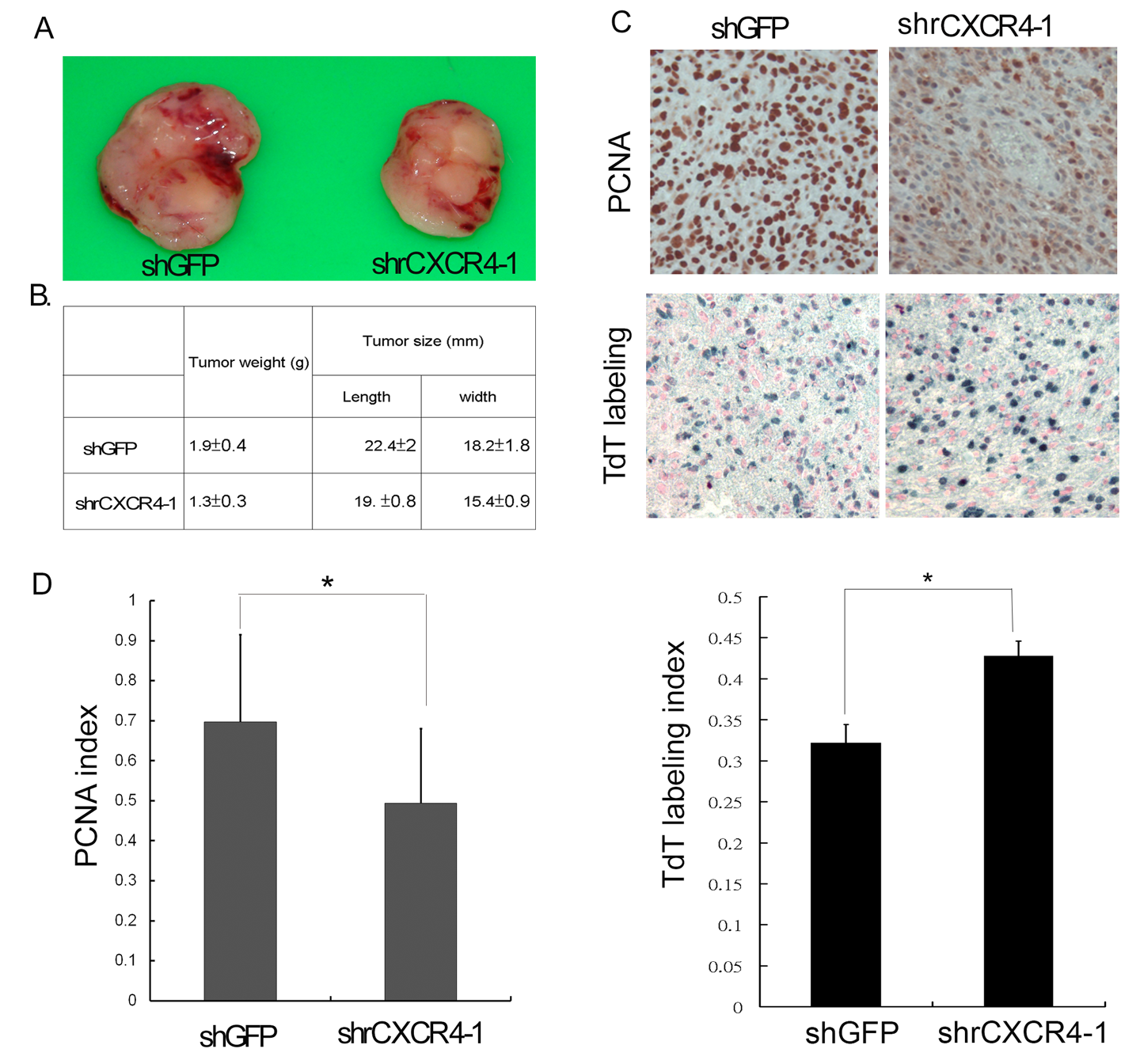

Supplement: Additional file 3: Figure S2 — Disruption of CXCR4 results in RG2 proliferation deficiency and the increase of apoptosis in vivo. To explore the effect of CXCR4 disruption in the tumorigenesis of GSC in vivo, shrCXCR4-1 cells and shGFP cells were subcutaneously injected into NOD-SCID mice. After 21 d, both shrCXCR4 and shGFP cells grew to tumor mass. Xenografts derived from shrCXCR4 showed a tumor mass size that was smaller than those derived from shGFP (A, B). Immunohistochemistry using anti-PCNA, a proliferating cell marker, was performed to explore the proliferating cells of sections obtained from shGFP and shrCXCR4 xenografts. The PCNA-positive population of xenografts derived from shGFP was 70% and significantly dropped to 50% of those derived from shrCXCR4 (C, D). PCNA index: PCNA positive cells (brown)/hematoxylin positive cells (blue). The results showed that there were fewer proliferating cells in the xenografts derived from shrCXCR4-1 than those in the xenograft derived from shGFP. The apoptotic cells were revealed by TdT labeling by using TACS2 TdT-blue label in situ apoptosis detection kit (Travigen, Inc. Cat. 4811-30-K). The result showed that the apoptotic population of xenografts derived from shGFP was 30% and significantly increased to 40% of those derived from shrCXCR4 (C TdT labeling, D TdT labeling index). TdT labeling index: TdT label positive cells (blue)/nuclear fast red positive cells (red). This observation suggested that CXCR4 plays an essential role in the proliferation of glioblastoma cells. *t test, P < 0.05. [file 1475-2867-13-85-S3.tiff]

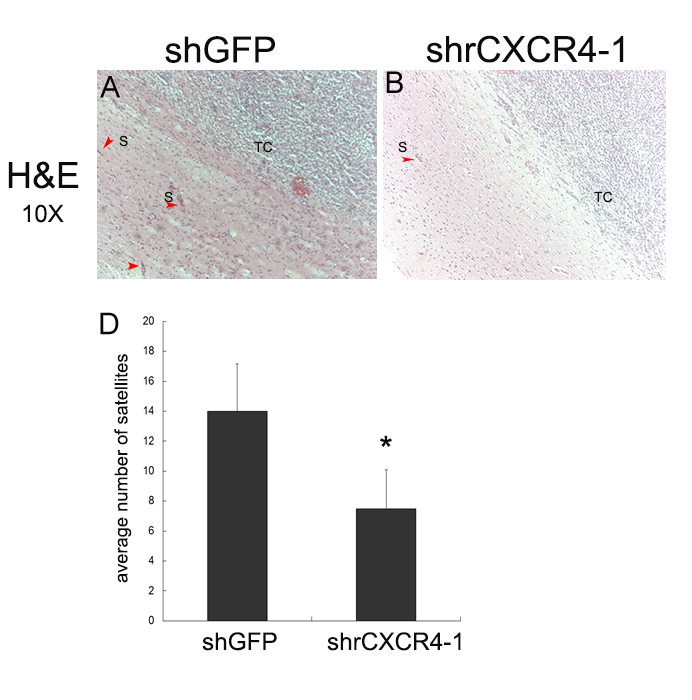

Supplement: Additional file 4: Figure S3 — Disruption of CXCR4 markedly reduced the number of satellites, which were defined as tumor foci with blood vessel and away from the tumor core (TC). H&E staining revealed a reduced number of satellites (arrow) in the intracranial grafts from shrCXCR4-1 RG2 (B) as compared with those from shGFP RG2 (A). (C) Average of number of satellites per xenografts, indicating less number of satellites in the xenografts derived from shrCXCR4 RG2 as compared with those derived from GFP RG2. Number of satellites was counted as the average of 6 adjacent sections of each grafts. Representatives are the average number of satellites from 5 xenografts derived from shrCXCR4 or shGFP RG2. [file 1475-2867-13-85-S4.tiff]
