# Supplementary material for: Disrupting the CXCL12/CXCR4 axis disturbs the characteristics of glioblastoma stem-like cells of rat RG2 glioblastoma
Source: Cancer Cell Int. 2013 Aug 21;13:85. doi: 10.1186/1475-2867-13-85 (PMC3765790; doi:10.1186/1475-2867-13-85)
Supplement: Additional file 2: Table S1 — A high level of CXCR4 is associated with the pathological status of glioblastoma. [file 1475-2867-13-85-S2.doc]

|  | CXCR4(low)  N= 22 | CXCR4 (high)  N= 47 | P value) |  |
| --- | --- | --- | --- | --- |
| Age(years) | 40.63±17.54 | 42.04±15.42 | 0.306 |  |
| Gender ratio (M/F) | 9/13 | 27/19 | 0.097 |  |
| Type  Normal  Malignant | 9  13 | 0  47 | <0.001 |  |
| Pathology  Astrocytoma  Glioblastoma  Pleomorphic glioblastoma  Glioblastoma multiforme | 9  4  0  0 | 22  12  2  10 | <0.001 |  |

**Table S1: High Level of CXCR4 is associated with the pathologic status of glioblastoma**

Statistical analysis of correlation between pathological status and CXCR4 levels was performed using the GraphPad Prism and Statistical Package for Social Sciences 15.0 (SPSS Inc., Chicago, IL, USA). Statistical methods included Analysis of variance (ANOVA). ANOVA was used in the case of comparison of multiple groups.
