# Supplementary material for: Disrupting the CXCL12/CXCR4 axis disturbs the characteristics of glioblastoma stem-like cells of rat RG2 glioblastoma
Source: Cancer Cell Int. 2013 Aug 21;13:85. doi: 10.1186/1475-2867-13-85 (PMC3765790; doi:10.1186/1475-2867-13-85)
Supplement: Additional file 5: Table S2 — Listed antibodies for immunoblot blot and immunohistochemistry. [file 1475-2867-13-85-S5.doc]

**Table S2: Listed antibodies for Western Blotting and immunohistochemistry**

Western blot

| **Antibody** | **Host** | **Condition** | **Company** | **Cat. No** | **Location** |
| --- | --- | --- | --- | --- | --- |
| CXCR4 | rabbit | 1/2000 | abcam | ab7199 | Cambridge, MA |
| Nanog | rabbit | 1/1000 | abcam | ab106465 | Cambridge, MA |
| Oct_4 | rabbit | 1/500 | abcam | ab18976 | Cambridge, MA |
| Sox2 | rabbit mo | 1/1000 | Cell signal | 3579 | Danvers, MA |
| p-AKT | rabbit | 1/1000 | Cell signal | 9271 | Danvers, MA |
| AKT | rabbit mo | 1/1000 | Cell signal | 9272 | Danvers, MA |
| p-ERK | mouse mo | 1/1000 | Cell signal | 9106 | Danvers, MA |
| ERK/1/2 | rabbit | 1/1000 | Cell signal | 9102 | Danvers, MA |
| SDF-1 | rabbit | 1/200 | Santa Cruz | SC-28876 | Burlingame, CA |
| LIN-28 | rabbit | 1/1000 | Santa Cruz | SC-67266 | Burlingame, CA |
| anti-rabbit | goat | 1/200000 | Jackson | 111-035-003 | Baltimore, MD |
| anti-mouse | goat | 1/200000 | Jackson | 115-035-062 | Baltimore, MD |

Immunohistochemistry

| **Antibody** | **Host** | **Condition** | **Company** | **Cat. No** | **Location** | **Retrieval(0.1M Sodium Citrate)** |
| --- | --- | --- | --- | --- | --- | --- |
| CXCR4 | rabbit | 1/500 | abcam | ab2074 | Cambridge, MA | 100℃, 10min |
| VEGF | mouse | 1/200 | abcam | ab68334 | Cambridge, MA | 100℃, 20min |
| CD31 | rabbit | 1/50 | abcam | ab28364 | Cambridge, MA | 121℃,15lb, 15min |
| CXCL12* | *protein | | R&D | 460-SD | Minneapolis, MN |  |
