# Supplementary material for: Disrupting the CXCL12/CXCR4 axis disturbs the characteristics of glioblastoma stem-like cells of rat RG2 glioblastoma
Source: Cancer Cell Int. 2013 Aug 21;13:85. doi: 10.1186/1475-2867-13-85 (PMC3765790; doi:10.1186/1475-2867-13-85)
Supplement: Additional file 6: Table S3 — Listed primers for RT-PCR. [file 1475-2867-13-85-S6.doc]

**Table S3 : List of Primers for RT-PCR**

| **Item** | **Sequence** | **Length (bp)** |
| --- | --- | --- |
|  |  |  |
| rOct 171 | 5'-CCT CgA ACC Tgg CTA AgC TTC C-3' | 200 |
| rOcT 372 | 5'-CCA CTC gTg CTC CTg CCT gg-3' |  |
|  |  |  |
| rSox2 501 | 5'-CAT gAA TgC CTT CAT ggT gTg g-3' | 225 |
| rSox2 726 | 5'-CCT TCT TCA TgA gCg TCT Tgg-3’ |  |
|  |  |  |
| rNanog 237 | 5'-CCT AgT TgT gAg gAA gCA TCg-3' | 202 |
| rNanog 439 | 5'- GAC CAG ACA GCT TTA GCT TGG-3’ |  |
|  |  |  |
| rCD133 661 | 5'-CTT AGC CAT CTC CCT CTT GC-3' | 225 |
| rCD133 886 | 5'-CTG TCC AGG TCT GAG AAT GC-3' |  |
|  |  |  |
| rMGmt 96 | 5'-AAT ACA Cgg TgT Tgg ACA gC-3' | 221 |
| rMGmt 317 | 5'-CCT CTg Tgg CTg CAg gTT Cg-3' |  |
|  |  |  |
| rALdh 72 | 5'-ACT TgA AgA TTC AAC ACA CC-3' | 201 |
| rAldh 273 | 5'-CTC TgA AgC ATC CAT ggT gC-3' |  |
|  |  |  |
| b1integrin 276 | 5'-ggA gAA TgT ATA CAA gCA gg-3' | 198 |
| b1integrin 472 | 5'-CCT TTg CTg CgA TTg gTg AC-3' |  |
|  |  |  |
| r CXCR4 41 | 5’-ggT CTg gAg ACT ATg ACT CC-3’ | 241 |
| r CXCR4 282 | 5’-AAg TAC CAg TCA gCC ATg gC-3’ |  |
|  |  |  |
| MELK 179 | 5’-gAC Agg Tgg CTT TgC AAA gg-3’ | 194 |
| MELK 363 | 5’-TCT TTg TCT CTA GCA CAT gg-3’ |  |
|  |  |  |
| rGAPDH 153 | 5'-ggA CAT TgT TgC CAT CAA CgA CC-3' | 369 |
| rGAPDH 522 | 5'-CTA AgC AgT Tgg Tgg TgC CC-3' |  |
|  |  |  |
| rMMP9 18 | 5’-CCT gCT CCTggT gCT CCT gg-3’ | 342 |
| rMMP9 360 | 5’-gAT ggT gCC ACT TgA ggT Cg-3’ |  |
|  |  |  |
| MMP2 142 | AgA gTT ggC AgT gCA ATA CC | 234 |
| MMP2 376 | TgT gAT CTg ATT CTT gTC C |  |
|  |  |  |
| VE-cadherin 753 | 5'-AAg ACA TCA ATg ACA ACT TCC-3' | 453 |
| VE-cadherin 1211 | 5'-ggA Tgg AgT ATC CAA TgC T-3' |  |
|  |  |  |
| ANGPT1 487 | 5’-CCA TTC TgA CTC ACA TAg g-3' | 164 |
| ANGPT1 651 | 5'-TTC CAC AAT gTA ATT CTC AAg-3' |  |
|  |  |  |
| ANGPT2 48 | 5'-gCT gTg ATC TTg TCT Tgg C-3' | 272 |
| ANGPT2320 | 5'-TTC TTC ATg TTg TCC Tgg-3' |  |
|  |  |  |
| rABCB1b 273 | 5'-CAG AGA CCC GCA TTC TGC CGA GC-3' | 227 |
| rABCB1b 500 | 5'-TCA AAC CAG CCT ATC TCC TG-3' |  |
|  |  |  |
| ABCB1b 326 | 5’-CAC AGA CCG TCA GCG ACA GC-3’ | 224 |
| ABCB1b 550 | 5’- GGC TCA CAG ATG ACG TCT CC-3’ |  |
|  |  |  |
| VEGF 889 | 5'-CCT Cgg TTC CAg Aag ggA gAg g-3' | 507 |
| VEGF 1396 | 5'- CAC ATC TgC TAT gCT gCA gg-3' |  |
|  |  |  |
| ABCG2 191 | 5'-TAT AAT GGG ATC ATG AAA CC-3' | 142 |
| ABCG3 333 | 5'-CGA AGA GCT GAG AAC TG-3' |  |
| rCXCR4 sh RNA sequence | 5'-GAT CCGCTA CTC CGA AGA AGT AGGGTT CAA GAGACC CTA CTTCTTCGGAGTAGT TTT TTGGAA A-3' |  |
|  | 5'-AGCTTTTCCAAAAAA CTA CTC CGA AGA AGT AGG GTC TCT TGA ACC CTA CTTCTTCGGAGTAGCG-3'. |  |
| GFP sh RNA sequence | 5'-GATCC GGTTATGTACAGGAACGCA TTCAAGAGA TGCGTTCCTGTACATAACC TTTTTGGAAA-3' |  |
|  | 3’-G CCAATACATGTCCTTGCGT AAGTTCTCT ACGCAAGGACATGTATTGG AAAAACCTTTTCGA-5' |  |
